# Motor Racing and Health

**Published:** 1903-06-06

**Authors:** 


### The Hospital.

A Journal of

The Medical Sciences and Hospital Administration.

Edited by Sir Henry Burdett, K.C.B.

Vol. XXXIV.—No. 871. [June 6, 1903.

### Motor Racing and Health.

Few things are more curious, to the dispassionate observer of the daily currents of human thought, than the sudden development, under the influence of some event which weighs upon the popular imagination, of opinions or demands entirely opposed to those which had previously been in force, and which had rested, or appeared to rest, upon a long course of experience and of judgment. Nothing is more certain than that all sports which require the exercise of strength, skill, and courage are associated with an element of danger which can never be entirely eliminated, and which, from time to time, inevitably demands its toll of victims. Shooting, hunting, racing, yachting, cycling, football, even cricket, collectively furnish an unfailing supply of accidents and of fatalities to the columns of the daily press; and yet, in spite of them, no one has been found hardy enough to dispute the assertion that Waterloo was won upon the playing-fields of Eton, or that the habitual practice of such games or sports tends to the production and maintenance of a high standard of manliness throughout the country. To the spirit of knighthood, as Scott wrote:

> "If a path be dangerous known,
> The danger's self is lure alone."

But still, notwithstanding this, which we would venture to describe as the established view of things, the mere fact that a motor race, conducted on the Continent in a manner which set every dictate of the most ordinary prudence at defiance, was attended by fatalities which may almost be said to have been asked for by mismanagement, has been made the basis of an outcry against the forthcoming motor race in Ireland, a race which has been deliberately sanctioned by the Legislature and will be surrounded by every safeguard which experience can supply. We have not the slightest intention of denying that motor racing is what old ladies of a benevolent turn of mind would call "dangerous," but we affirm that a willingness to face danger has made our country what she is, and that any decadence in this respect would imply a danger more considerable still—the danger of the loss of her position among nations. We have no sympathy with foolhardiness, and very little compassion for the fate of those who suffer its inevitable consequences; but the residual element of danger in a sport for which prudence has done its best is something which should be recognised for what it is, and faced with the courage which the circumstances may require. All who engage in such sports may, if they so please, fully test their fitness for them before accepting their more formidable responsibilities, and may draw back in time if they find themselves unequal to demands for which it is a duty to be prepared.

We fear it is only too true that motor racing as a sport will have but little influence, one way or the other, upon the popularising of such carriages for the more ordinary purposes of life. A racing motor differs from a road motor at least as much as a wager boat does from a dinghy, or the successive *Shamrocks* from a Thames lighter—not only, that is, in mere appearance, but fundamentally in the principles of its construction; and hence it follows that the most brilliant success in the construction of cars designed for speed may have very little tendency to promote the improvement of such as are designed for the conveyance of passengers or merchandise. The racing, again, must always be an extremely costly amusement, requiring long distances of prepared tracks, machines on which almost unlimited sums of money may be spent, and very elaborate precautions for the safety of spectators. But, even in spite of these difficulties, it seems to us likely to hold its own. No one who has not experienced it can form any adequate idea of the intense exhilaration produced by being conveyed through the air at very high rates of speed. The contrast in this respect between the ordinary pace of a cab horse and that of a fast trotter is significant enough, and the footplate of an express locomotive is to the trotter what the trotter is to the cab horse; while the pride with which an engine driver regards his engine, or the affection with which he speaks of it as "she," are quite sufficient to explain the love of the motor owner for his machine and for the sensations incidental to making "her" do her best. We have seen in various journals some very bad specimens of nonsense about the tendency of motor driving to produce some mysterious form of mental disease, which exists only in the imaginations of the gentlemen whom Charles Dickens was wont to describe as "mad doctors." There is no essential difference, of course, between driving a road motor and driving a locomotive; and the necessity of dealing with a larger and variable amount of other traffic, in the former case, is practically counterbalanced by the greater ease with which the pace can be controlled, and by the rapidity with which the machine, being unhampered by the momentum of a train behind it, can be brought to a stand. Both pursuits require attention, and in both attention, being only a human faculty, will sometimes flag. The Great Western driver who, a year or two ago, ran past the signals at Slough, will now and then have his counterpart on a road motor, and now and again some other user of the road will have good reason for complaint, but will, we hope, generally be able to sign himself, as a gentleman did last week in writing to the *Times*, as "Dei Gratiâ Superstes." We look forward with much hope to the promised legislation concerning road motors which will relieve them from their present liability to be perpetually harassed by foolish justices and by police spies, while at the same time it will afford to the general traffic all necessary protection, and will enable the new carriage, which must unquestionably in time supersede all others, to develop itself along such lines as the public convenience may require.

